# Elucidation of Mechanisms by Which Microplastics (PET) Facilitates the Rapid Growth of Benthic Cyanobacteria and Toxin Production in Aquatic Ecosystems

**DOI:** 10.3390/metabo15060383

**Published:** 2025-06-09

**Authors:** Rashid Mir, Shrooq Albarqi, Wed Albalawi, Ghaida Alanazi, Shouq S. Alsubaie, Razan I. Alghaban, Hanadi Saud Alanazi, Nora Taleb Alsharif, Manal M. Aljammaz, Nouf Faisal Alghabban, Wafaa Seluman Alhwiti, Alaa Albogmi, Faras Falah Alblwi

**Affiliations:** 1 Prince Fahd Bin Sultan Chair for Biomedical Research, University of Tabuk, Tabuk 71491, Saudi Arabia; aabogmi@hotmail.com; 2 Department of Medical Lab Technology, Faculty of Applied Medical Sciences, University of Tabuk, Tabuk 71491, Saudi Arabia; albarqishrooq@gmail.com (S.A.); wed_abdullah@outlook.sa (W.A.); ghaida3331@gmail.com (G.A.); shoogalhawwas@gmail.com (S.S.A.); razanialghaban@gmail.com (R.I.A.); hanadi.saud@hotmail.com (H.S.A.); nora.taleb96@gmail.com (N.T.A.); m.m.aljammaz@hotmail.com (M.M.A.); nf2626@gmail.com (N.F.A.); wafa-al-hwiti@hotmail.com (W.S.A.); fares.0252@hotmail.com (F.F.A.)

**Keywords:** polyethylene terephthalate (PET), microplastics, PET leachates, benthic cyanobacteria, plastic-associated chemicals

## Abstract

Polyethylene terephthalate (PET) is one of the most frequently used synthetic polymers and it plays a major role in plastic pollution in aquatic environments. As PET undergoes environmental degradation, it sheds microplastics and chemical leachates, which have an effect on microbial communities, including benthic cyanobacteria. This review focuses on the molecular processes by which PET microplastics and their associated leachate affect the growth, physiological performance, and ecological performance of benthic cyanobacteria. We explore how PET-derived compounds serve as carbon and energy sources or signaling molecules, possibly affecting photosynthesis, nitrogen fixation, or stress response pathways through changes in gene expression. Moreover, the function of PET leachates as environmental modulators of microbial community structure, generators of reactive oxygen species (ROS), and disruptors of hormonal and quorum sensing networks are also outlined. Knowledge of these interactions is essential for the evaluation of the wider ecological risks resulting from plastic pollution and the likelihood of cyanobacterial blooms in PET-polluted environments. This review synthesizes evidence on how PET microplastics and leachates act as carbon sources and stressors, modulating gene expression to promote benthic cyanobacterial growth and toxin production, potentially exacerbating ecological risks in polluted aquatic systems.

## 1. Introduction

Plastic pollution has emerged as a critical environmental challenge, driven by the escalating global production of plastic materials and inadequate waste management practices. Microplastics (MPs), defined as plastic particles smaller than 5 mm, have attracted growing concern due to their environmental persistence, widespread distribution, and potential to transport hazardous chemicals in aquatic ecosystems [1]. These particles can originate directly as small fragments (primary MPs) or result from the degradation of larger plastic debris (secondary MPs). Once released into the environment, MPs interact with aquatic organisms, disrupting ecological processes and potentially causing toxic effects, such as endocrine disruption and developmental abnormalities [2]. Wastewater treatment plants (WWTPs), while designed to remove contaminants, often serve as both reservoirs and pathways for microplastics, with effluent concentrations reported as high as 447 particles per liter [3]. The 447 particles per liter found in a European tertiary treatment plant is a high-end result, while typical concentrations are much lower, ranging from 0.1 to 447 particles per liter depending on plant design, treatment methods, and what enters the plant [4,5]. For example, primary treatment plants may release more than 10,000 microplastics per liter, whereas advanced filters such as membrane bioreactors, can lower the number of microplastics in the water to less than one per liter [6,7]. Such concentrations are a concern for the environment, since even a small amount (1–10 particles per liter) can build up in water, exposing organisms over a long period. MPs at a concentration of 10 particles per liter can cause oxidative stress in cyanobacteria and may increase the rate of toxin production, like microcystins, by 1.5 times [8,9]. The environmental importance of these concentrations is highlighted when they adsorb persistent organic pollutants such as polychlorinated biphenyls which increases their effects and toxicity on aquatic life [10].

Among the various polymer types, polyethylene terephthalate (PET) stands out for its extensive usage in consumer goods, particularly in packaging and textiles and for its environmental persistence. PET is commonly found in the near-bottom waters and surface sediments of freshwater systems, where it can account for up to 50% of detected microplastics [11]. Due to its relatively high density and resistance to biodegradation, PET tends to accumulate in benthic environments. Over time, environmental aging processes such as UV radiation, mechanical abrasion, and microbial action alter the surface chemistry of PET, enhancing its hydrophilicity and reactivity. These changes increase its ability to adsorb pollutants, promote biofilm formation, and influence the surrounding microbial communities. Notably, aged PET can release chemical leachates, further contributing to its ecological impact [12].

**Figure 1:**
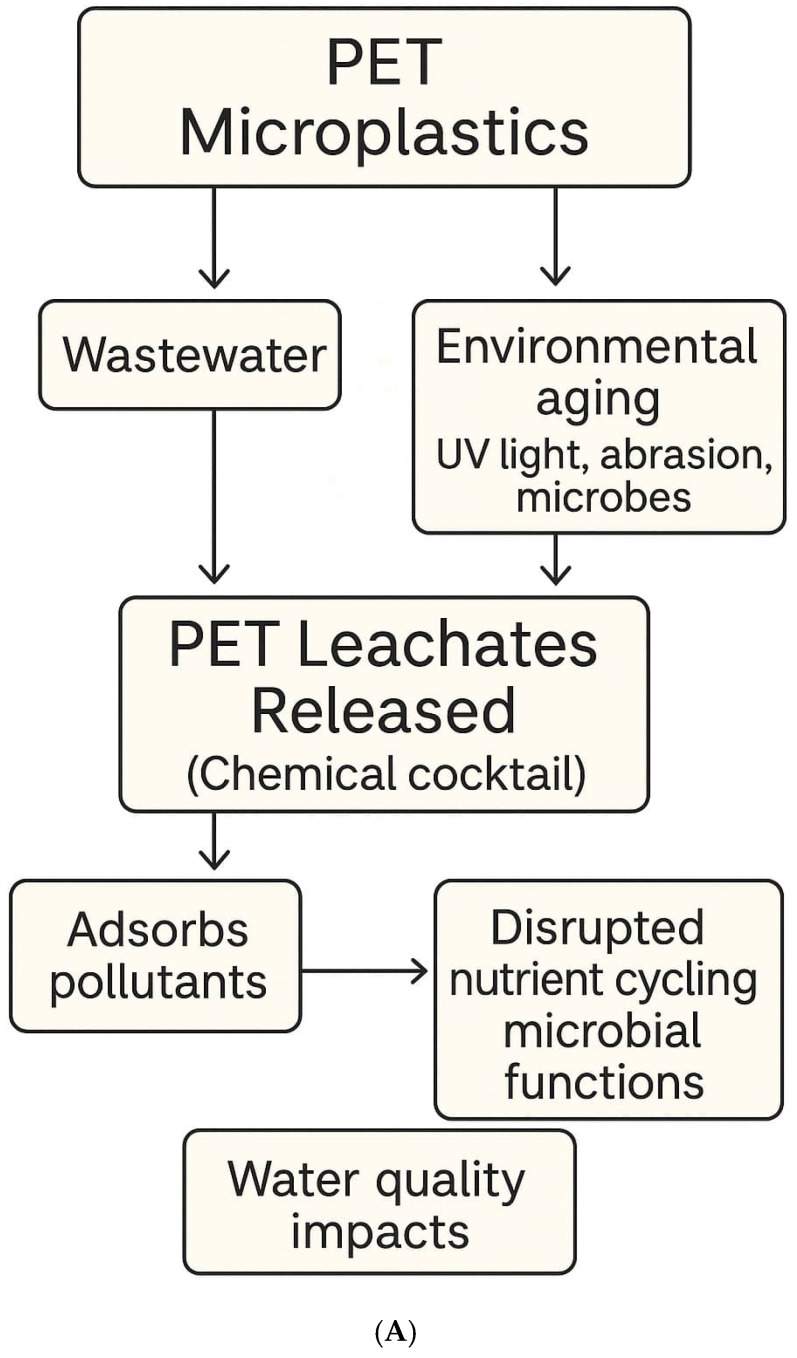

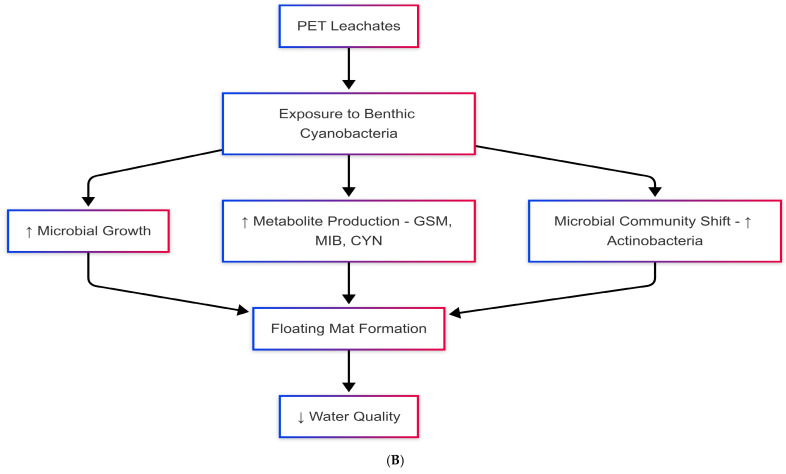
(**A**): **PET microplastic degradation and leachate formation**: The schematic illustrates the pathway through which polyethylene terephthalate (PET) microplastics enter aquatic environments, undergo environmental aging (UV radiation, abrasion, and microbial degradation), and release chemical leachates. These leachates also adsorb environmental pollutants and disrupt microbial and nutrient cycling processes, initiating ecological impacts. (**B**): **Microbial and ecological response to PET leachates:** This Figure 1B. summarizes the downstream biological responses of benthic cyanobacteria to PET-derived leachates. These interactions promote microbial growth, increase metabolite production (e.g., geosmin [GSM], 2-methylisoborneol [MIB], cylindrospermopsin [CYN]), and shift microbial communities—particularly increasing *actinobacteria*. These effects lead to floating mat formation and impact water quality.

Cyanobacteria are a group of photosynthetic microorganisms that inhabit diverse ecosystems, including freshwater, marine, and terrestrial environments. While much attention has been devoted to planktonic (free-floating) cyanobacteria and their role in harmful algal blooms, benthic **cyanobacteria**—those that grow attached to substrates such as sediments and rocks—remain comparatively underexplored [13] as depicted in Figure 1A. Under favorable conditions, such as elevated temperatures, nutrient enrichment, and light availability, benthic cyanobacteria can proliferate and form dense mats. These mats, composed of filamentous cyanobacterial species embedded in a matrix of extracellular polysaccharides, often trap oxygen bubbles during photosynthesis, causing fragments to rise to the water surface as floating mats [14].

Benthic cyanobacteria are ecologically and toxicologically significant. Species such as *Oscillatoria* and *Pseudanabaena* are known to produce harmful secondary metabolites, including cylindrospermopsin (CYN), geosmin (GSM), and 2-methylisoborneol (2-MIB) [15] as depicted in Figure 1B. These compounds can cause organ toxicity and produce unpleasant tastes and odors at extremely low concentrations, compromising drinking water quality and aquatic ecosystem health [16]. In addition, benthic mats can harbor actinobacteria, which are also capable of producing odor compounds [16]. Detection of these compounds is challenging due to limited monitoring and the difficulty in culturing benthic microbial communities. However, recent advances in molecular tools such as quantitative PCR targeting biosynthetic genes for GSM and MIB offer promising solutions for early detection [17].

Studies have identified microplastics, including PET, within benthic cyanobacterial mats, indicating complex interactions with microbial communities [18,19]. However, most research to date has focused on how microplastics affect planktonic cyanobacteria, with minimal attention paid to benthic forms [20]. Given the structural and physiological differences between planktonic and benthic cyanobacteria, such as their size, filamentous morphology, and adaptability to low light, the effects of microplastics on benthic cyanobacteria may differ significantly. For example, *Oscillatoria* filaments can exceed 180 μm in length, compared to the ~3.6 μm diameter of the unicellular *Microcystis aeruginosa*. These structural differences may influence how microplastics interact with benthic cyanobacteria, particularly in terms of particle adhesion and shading effects [21].

The world faces a crucial environmental crisis because of increasing plastic material production alongside deficient waste management practices. The scientific community now closely watches microplastics (MPs) since these plastic fragments measuring below 5 mm have proven to persist in the environment as they spread extensively throughout aquatic ecosystems while serving as carriers of harmful chemicals [22]. Plastic material breaks down into two types of particles: primary small fragments and secondary fragments that develop from disintegrating larger plastic items [23]. Microplastics that enter environmental systems disrupt aquatic life processes and generate toxic effects including endocrine disruption and developmental abnormalities among organisms [24]. WWTPs which are meant to eliminate contaminants have been identified as both plastic material reservoirs and transmission pathways and scientists have measured effluent microplastics at 447 particles per liter [25]. Nitrification, alongside denitrification, methanogenesis, and other microbial functions, becomes affected by MPs during nutrient cycling operations. PET microplastics constitute around half of the microplastics detected in fresh water near-bottom waters together with surface sediments [26]. PET aggregates in benthic environments because it possesses high density combined with its resistance to biodegradation. PET materials become more hydrophilic and reactive after undergoing environmental aging through exposure to UV radiation as well as mechanical abrasion and microbial attacks over time [27]. The modification of PET by environmental aging processes enhances its capability to absorb pollutants while facilitating biofilm development and affecting the microbial populations in the surrounding area. The aging of PET materials enables the substance to release chemical leachates that worsen its environmental effects [28].

Photosynthetic cyanobacteria exist throughout different natural environments which include freshwater bodies as well as marine areas and terrestrial locations. Benthic cyanobacteria that dwell on sediments and rocks receive less scientific investigation despite their importance during harmful algal blooms compared to planktonic cyanobacteria [13]. Temperature increases together with nutrients and light exposure enable benthic cyanobacteria to multiply into thick mats. The photosynthetic process of filamentous cyanobacteria embedded in extracellular polysaccharides produces oxygen bubbles that create floating mat fragments that rise to the water surface [29]. The presence of benthic cyanobacteria represents a strong ecological and toxicological concern. The harmful secondary metabolites cylindrospermopsin (CYN) and geosmin (GSM) along with 2-methylisoborneol (2-MIB) are produced by benthic cyanobacteria species *Oscillatoria* and *Pseudanabaena* [30]. The presence of these compounds induces organ damage while producing offensive tastes and smells at very small amounts which degrades water quality and harms aquatic ecosystems. The mats found beneath the water surface contain actinobacteria that generate odor compounds [31].

Benthic cyanobacterial mats contain microplastics, indicating potential relationships between pollutants and microbial communities according to recent research findings. Research about microplastics primarily investigates their effects on planktonic cyanobacteria while benthic forms receive little attention [32]. The physical characteristics and cellular organization of benthic cyanobacteria differ substantially from planktonic cyanobacteria through their size and filamentous shape and their capacity to survive under low-light conditions. So, their response to microplastics exposure will be distinct. The filamentous *Oscillatoria* extends beyond 180 μm whereas the *Microcystis aeruginosa* exists as unicellular units with a diameter of ~3.6 μm [33]. The different structures of benthic cyanobacteria would affect their interactions with microplastics particularly concerning how particles bind to the cells and how light is blocked [34]. The biological effects of leachates produced by aged PET microplastics appear to be unique. Studies present conflicting results about microplastic leachate impacts on planktonic cyanobacterial growth because the effects depend on which polymer is used and its concentration level. The diverse and specific reactions of benthic cyanobacteria demonstrate why researchers must direct their studies toward these organisms. Studies need to investigate the unidentified molecular mechanisms which control benthic cyanobacteria responses toward PET microplastics together with their leachate substances [35].

This study investigates the molecular pathways that PET microplastics and PET-derived leachates use to regulate benthic cyanobacteria development alongside alterations to metabolite output. Through research synthesis and focused gap analysis, our work strives to develop environmental and molecular knowledge about the sediment–water interface where benthic cyanobacteria and PET microplastics are becoming more common. Knowledge of plastic–water interactions remain essential for assessing freshwater ecosystem risks while developing proper water quality management strategies during the global plastic pollution crisis. Despite growing concern over microplastic pollution, the specific molecular and ecological mechanisms by which PET microplastics and their leachates affect benthic cyanobacteria remain underexplored. This review aims to address this gap by synthesizing recent evidence on PET-induced physiological responses, including oxidative stress, toxin production, and quorum sensing disruptions in benthic cyanobacterial communities.

## 2. Polyethylene Terephthalate (PET) in Aquatic Environments

### 2.1. Sources and Distribution of PET Microplastics

PET is a popular thermoplastic polymer that is valued for its strength, lightness, transparency, and resistance to chemicals. So, it is used in beverage bottles, food packaging, and synthetic materials [36]. The material longevity and extensive usage patterns of PET have resulted in substantial contribution to worldwide plastic contamination especially within aquatic settings [37]. PET microplastics are formed by microbeads and pre-production pellets, as well as from the degradation of larger PET items due to friction, sunlight and bacteria [38]. PET ends up in freshwater mainly when people improperly dispose of it, when runoff from cities contains it, when landfills leak, or when it is discharged from factories [38,39]. Because of ineffective wastewater filters, these treatment plants often release PET microplastics even though their goal is to clean the water. These byproducts are a main cause of PET contamination in rivers and lakes that may eventually reach the sea [40]. It has been found that PET microplastics are spreading around the world, with more than 80% of tested tap water samples showing their presence and major concentrations in bottled water [41,42]. PET particles have been detected in sediments from the Tibetan Plateau, the Arctic Ocean, and all around the Ganga River basin in India—both in cities and rural areas [43].

**Figure 2:**
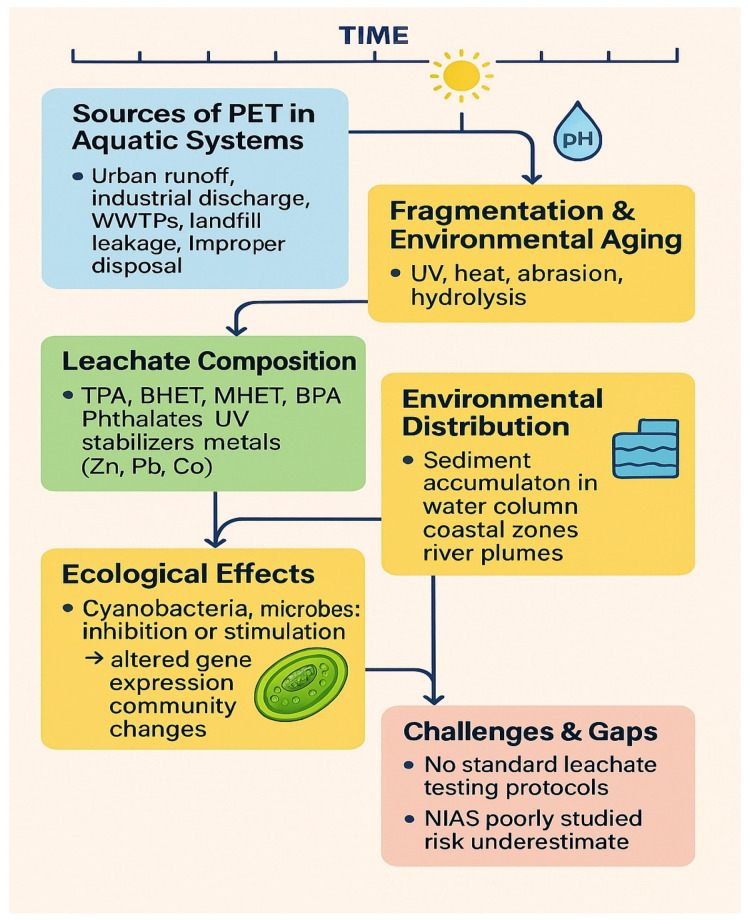
Fate and impact of PET in aquatic systems. Figure showing PET fragmentation, leachate formation, and ecological effects on benthic zones, including microbial and contaminant interactions.

PET shows a distinctive characteristic that guides its distribution through aquatic systems because its density value at 1.38 g/cm^3^ makes it sink rather than buoy as depicted in Figure 2. The density characteristics of PET lead to its accumulation at benthic zones near the ocean floor because these particles sink to bottom waters and sediment deposits where they remain for long periods [11,44]. Because of the sedimentation, PET particles become stable and can interact with microbes in the benthic community, mainly with cyanobacteria. These processes may change the way biogeochemical cycles function, increase activity among microbes, and boost the movement of pollutants [45,46]. The high surface-area-to-volume ratio and surface chemistry of PET microplastics facilitate the adsorption of heavy metals, persistent organic pollutants (POPs), and endocrine-disrupting chemicals [47], which may be bioavailable to aquatic organisms upon ingestion, amplifying their ecological effects [1,48].

### 2.2. Environmental Aging and Surface Modification of PET

PET is exposed to various environmental aging processes after being introduced to aquatic systems that cause significant modifications to its surface features as well as chemical composition and biotic interactions [49]. The aging processes of PET occur through multiple factors that include ultraviolet (UV) radiation exposure together with temperature changes, mechanical wear, hydrolysis, and biological activity. The physical breakdown of PET along with its increased surface reactivity occurs through these aging processes which results in greater environmental impact [50].

One year of exposure to outdoor UV light on PET fragments (3.525 µm) showed that the material suffered cracks and edge breaks, resulting in a 1.3% loss in weight. On the other hand, indoor-aged PET experienced very little weight loss (only about 0.40%), indicating that it degrades more slowly without sunlight. Hydroperoxides are generated on the surface of plastics when ester groups are oxidized by UV light, increasing roughness and supporting microbial growth. Because of these changes, PET is more likely to attract water and react with benthic cyanobacteria, encouraging the formation of biofilm. When a material ages outdoors for a long time, it gains new functional groups such as those shown by an extra peak at 2345.44 cm^−1^ on the FTIR that can boost the ability to trap heavy metals. When PET is modified in this manner, it is more able to transport contaminants to aquatic ecosystems, which can affect the behavior of cyanobacteria [51].

When surfaces suffer photodegradation, hydroxyl and carbonyl groups are formed, increasing both the hydrophilic properties and roughness of the surface, making it easier for microbes to attach and create biofilms [52]. Because of mechanical weathering, PET degrades to microplastics (less than 5 mm) and nanoplastics (less than 100 nm), making them more likely to move, react, and enter the tissues of aquatic organisms [53]. In nature, hydrolytic breakdown is not common, but it happens at temperatures above 67–80 °C, where PET monomers such as TPA, ethylene glycol, BHET, and MHET are released [54,55]. Microorganisms that live in water will face alterations in their metabolic pathways while their community structure changes due to the leaching compounds.

With age, PET may start to host many different microbes, for example, cyanobacteria, heterotrophic bacteria, and fungi [56]. The aged and roughened PET substrate serves as an effective platform for microbial cell attachment which includes cyanobacteria heterotrophic bacteria along with fungi [56]. The biofilms formed by microbes can boost polymer degradation through the production of PETase enzymes together with cutinases [57]. The availability of PET-derived leachates functions as carbon sources while toxic degradation products create selective pressure which results in changes in microbial community composition [58]. Aged PET particles contain the ability to discharge various chemical additives and environmental degradation products. PET-leachate research has identified the presence of phthalates together with aldehydes (including acetaldehyde and formaldehyde) and non-intentionally added substances (NIAS) [11]. The substances released into the environment from plastic materials demonstrate toxicity and endocrine disruption and mutagenic properties that affect aquatic life based on their exposure duration and concentration levels. Leachates demonstrate different impacts on cyanobacteria photosynthetic microorganisms since some research shows inhibition but other studies demonstrate growth promotion through low concentrations of leachates [59]. The various transformations PET undergoes in the environment do not decrease its harmful nature. Older and broken PET microplastics show higher ecological reactivity when compared to new unaltered ones [60]. The environmental aging combined with surface modifications of PET microplastics intensifies their ecological threats present in aquatic environments [61]. The aged microplastic particles function as enduring physical contaminants while maintaining chemical activity which disrupts microbial ecosystems and modifies biogeochemical processes and enables toxic substance distribution throughout ecological compartments [62]. Accurate assessment of PET pollution’s permanent effects requires a comprehensive understanding of these transformations to create effective mitigation approaches [63].

### 2.3. PET Leachates: Composition and Environmental Relevance

The thermoplastic polymer PET demonstrates durability together with lightweight properties and biodegradation resistance, making it suitable for beverage bottles, textiles, and packaging materials [64]. The commercial usefulness of PET stems from its key properties but these same characteristics create environmental stability and make its breakdown process complicated. PET products that age and degrade in aquatic ecosystems produce both micro- and nanoplastics, physical fragments, and various chemical compounds known as leachates [65]. The leachates result from physical, chemical, and biological degradation mechanisms and function as a strong environmental contaminant source that typically goes unnoticed. The leachates from PET consist of intentionally added substances (IASs) such as plasticizers and stabilizers and processing aids which manufacturers add during production and non-intentionally added substances (NIASs) that arise from thermal degradation photochemical reactions or microbial metabolism [66]. These additives gradually move from the polymer matrix toward surrounding aquatic environments as they face environmental exposure to sunlight and temperature changes and microbial colonization. PET leachates remain invisible even though their biological activity surpasses that of particulate microplastics due to their chemical makeup, reactivity, and high-solubility characteristics [67].

The composition of PET leachates is highly variable and influenced by the specific polymer formulation, degree of environmental aging, and physicochemical conditions of the surrounding medium. Analytical studies have identified a multitude of compounds released from PET under simulated and natural degradation scenarios [68]. Among the most prevalent constituents are monomeric degradation products, such as terephthalic acid (TPA), bis(2-hydroxyethyl) terephthalate (BHET), and mono(2-hydroxyethyl) terephthalate (MHET). These result from the hydrolytic cleavage of the ester bonds in the PET backbone and are particularly prominent under elevated temperatures and alkaline pH conditions [69]. In addition to these hydrolysis by-products, PET leachates are known to contain a wide range of plastic additives and auxiliary chemicals, including phthalates (e.g., DEHP, DBP, and DIBP), bisphenol A (BPA), nonylphenol, benzothiazole, acetophenone, phthalide, and various benzotriazole-type UV stabilizers [70,71,72,73]. Flame retardants, such as polybrominated diphenyl ethers (PBDEs) and residual metal catalysts like zinc, lead, cadmium, cobalt, and manganese, have also been detected in PET leachates. Some of these metals originate from manufacturing processes, while others may be absorbed from surrounding environments due to PET’s capacity to adsorb and concentrate external contaminants onto its surface [74].

Various environmental conditions control the compound release rate. The leachate formation rate significantly increases when hydrolysis operates in alkaline waters with a pH value of 11 compared to acidic or neutral conditions. Temperature increases boost molecular diffusion which leads to better lower-molecular-weight additive release. UV radiation exposure causes photochemical breakdown which produces chain scission reactions and generates oxygenated functional groups that make the surface more reactive and polar. These alterations help substances to continue releasing while simultaneously promoting microbial population growth [75,76]. Biofilms composed of bacterial and fungal microorganisms on PET surfaces use PETase and MHETase extracellular enzymes to degrade PET while producing soluble intermediate chemical products that dissolve into surrounding water. Multiple laboratory and field studies demonstrate the growing importance of PET leachate effects on the environment [77]. The chemicals released from PET materials show harmful biological impacts that affect many aquatic organisms at minimal environmental dosages. PET leachates demonstrate toxic effects on microalgae and cyanobacteria primary producers through their ability to damage photosystem II which leads to reduced photosynthetic efficiency and lower chlorophyll content and oxygen production impairment [78,79]. Short-term exposure to PET leachates leads to transcriptional changes that affect genes related to oxidative stress response and detoxification and metabolic regulation according to transcriptomic analysis [80]. The damage to photosystem II (PSII) in microalgae and cyanobacteria by PET leachates is caused by certain chemicals and their actions. The main reason for PSII harm is the presence of bisphenol A (BPA) and phthalates, such as di(2-ethylhexyl) phthalate (DEHP), in leachate [81,82]. When exposed to light, these compounds make reactive oxygen species, such as superoxide and hydrogen peroxide, that damage the D1 protein in PSII [83]. The presence of oxidative stress interferes with the electron transport chain, leading to a lower photochemical efficiency in PSII [84]. BPA interferes with the QB-binding site of the D1 protein which stops electron transfer and leads to photoinhibition, reducing chlorophyll a fluorescence and the production of oxygen under controlled conditions [85]. Phthalates also damage the thylakoid membranes which results in further problems for PSII by changing the pathways of lipid peroxidation, causing malondialdehyde levels to go up in the cells exposed to phthalates [86]. This results in photosynthesis dropping by 10–40% and studies have found that microalgae exposed to 10 mg/L PET leachates for 96 hours have a 10–40% reduction in carbon fixation [87]. The amount of BPA or DEHP in leachate is related to the severity of PSII damage. This can have ecological results because a drop in photosynthesis in primary producers may upset the balance of carbon and oxygen in water [88,89]. More work is necessary to measure the individual effects of leachate components and how they interact with PSII in different environments.

PET leachate exposure affects aquatic invertebrate barnacles, copepods, and bivalves by causing developmental arrest, decreased survival rates, swimming behavior problems, and activating cellular stress pathways through lysosomal membrane damage [90]. The endocrine-disrupting chemicals BPA and nonylphenol, among other PET leachate components, cause hormonal disruption and reproductive system abnormalities as well as liver damage and persistent toxic substance buildup in fish and higher trophic level organisms. The sense organs and behavioral responses of gastropods and amphibians experience disruption when they encounter low levels of PET leachates in their environment [91]. The toxic effects of PET leachates occur independently from other substances. PET leachates consist of various chemical compounds that produce their toxicological responses through intricate interactions between numerous components whose individual effects might either strengthen or cancel each other out. Risk assessment methods and regulatory evaluation face substantial difficulty because of this situation [92]. Standard ecotoxicological tests analyze individual chemical substances while ignoring the unique toxic properties which emerge when multiple components mix together in environmental leachates. Environmental monitoring programs use non-targeted analytical methods insufficiently to detect and characterize the unknown NIAS [93].

PET leachate compounds become more complex due to microbial transformations. After release into aquatic systems, these compounds are metabolized by bacteria, fungi, and protists, potentially resulting in by-products with distinct toxicity patterns compared to the original compounds [94]. Scientists have limited knowledge about these transformation pathways which environmental fate models typically do not include. The ecological impact of PET leachates continues to increase yet these substances remain unregulated because environmental policies predominantly focus on visible plastic particles [95]. The EU’s REACH and Water Framework Directive include BPA and specific phthalates in their regulatory framework yet they do not consider all the unidentified substances that exist in leachate samples [96].

Opinions are divided about the effect of PET leachates on cyanobacteria. Certain studies observe that oxidative stress and disrupted photosynthesis lead to reduced growth [97], while Yang et al. found that, at low levels of leachate (10 mg/L), PET in the leachate can provide extra carbon and thus promote growth [98]. Such differences might result from the composition of the leachate, the kind of cyanobacteria involved, or the environmental setting (pH and temperature). Leachate is more likely to come out under alkaline conditions which amplifies the toxicity, whereas neutral conditions tend to promote the breakdown of chemicals. Standard methods of preparing leachate and comparing benthic and planktonic cyanobacteria can help resolve these contradictions [99].

The differences in how PET leachates affect cyanobacteria are caused by many factors that still require more research. What chemicals are in PET leachate depends on the type of polymer, any additives, and the way it decomposes in the environment [92,100]. A case in point is that excess BPA or phthalates in leachates can trigger oxidative stress by boosting ROS which stops photosystem II and results in *Microcystis aeruginosa* growth inhibition. Low levels of organic compounds, such as low-molecular weight esters, might be used by some species for growth, according to Yang et al. [101]. The impact of leachate on living organisms depends on the additives, including plasticizers and stabilizers, that are present, and how many of them there are. The ways each species is built and functions play a role in making responses different [102]. *Microcystis aeruginosa*, a planktonic cyanobacteria, might be more easily damaged by oxidative stress since it stays buoyant and is exposed to light-produced ROS [103], while *Nostoc spheroids*, a benthic kind, may respond better to stress because they are adapted to living in sediments [104]. Also, how well organisms can detoxify and handle carbon can change their reactions to leachates [105]. Leachate effects are greatly affected by the environmental factors of pH and temperature. A pH over 8 in the environment leads to the faster release of toxic monomers and additives from PET which can make growth inhibition worse [106]. On the other hand, microbes may break down the components of leachate into less harmful forms when the pH is neutral (6.5–7.5) which might account for the growth promotion at low concentrations [95]. Warmer temperatures (around 30 °C) can promote ROS production in leachate which increases toxicity, while moderate temperatures (between 20 and 25 °C) may assist the conversion of organic compounds by cyanobacteria [107,108]. Because these factors are not always managed the same way in studies, the results are sometimes different.

As there are no set procedures for preparing leachate, it is very difficult to study benthic cyanobacteria which are key to understanding the effects of PET leachate on the environment. It is difficult to reproduce the growth conditions of benthic cyanobacteria, such as *Nostoc* and *Oscillatoria*, because they naturally form intricate mat structures in their environment [109]. While planktonic cyanobacteria are easy to culture in liquid, benthic species need materials like sediment and agar, as well as specific light and nutrient conditions, and their mats are easily broken under laboratory conditions [110,111]. The sampling of benthic cyanobacterial mats is difficult due to their mixture of microorganisms, EPS, and sediment. Common sampling approaches, including grab sampling and coring, may harm the mat, resulting in a 15–25% lower estimate of biomass or toxins compared to results from in situ measurements [112,113]. Detection of toxins, such as microcystins in benthic mats, is made difficult by their hiding in EPS or sediment, as this reduces the extraction efficiency of standard tests [114]. Furthermore, it is difficult for non-targeted analytical methods to detect BIAS in leachates that can cause toxicity [115]. The problems in research methods mean it is hard to compare studies and determine the real impact of PET leachates on benthic cyanobacteria.

Standard methods for preparing leachate (using the same PET size, time, and solvent) along with experiments on multiple cyanobacteria and different environments are necessary to resolve these discrepancies as depicted in Figure 2. They would allow us to understand when PET leachates support or hinder plant growth and what their impact will be on the environment.

The assessment of PET leachate environmental risk needs future research to establish standard preparation methods for leachates alongside realistic exposure models and complete toxicology testing that includes complex chemical mixture analysis. The understanding of leachate toxicity mechanisms will strongly depend on the combination of advanced bioassay testing and -omics methods with adverse outcome pathway (AOP) frameworks. PET leachates represent an ecologically active part of plastic pollution which contains numerous significant chemical substances. The biological disruption ability of leachates during their interaction with food web base components creates extensive worries about their enduring effects on aquatic ecosystems. The combination of high presence and powerful nature along with the undetectable characteristics of PET leachates necessitates increased scientific research and environmental policy attention particularly in sediment-abundant zones that expose benthic organisms to chemical exposure. To facilitate understanding, Table 1 summarizes the sources, aging processes, and leachate impacts of PET microplastics, highlighting their role as both physical and chemical stressors in aquatic systems.

## 3. Production of Cyanotoxins and Taste and Odor Compounds

The production of secondary metabolites by benthic cyanobacteria leads to severe impacts on freshwater ecosystem health and drinking water safety. Cyanotoxins form one group of metabolites that pose toxicity risks for animals and humans yet taste and odor (T&O) compounds belong to another group that negatively impacts water sensory qualities without toxicity effects [118].

### 3.1. Cyanotoxins

Different types of cyanotoxins appear in benthic cyanobacteria production because they consist of neurotoxins, hepatotoxins, and cytotoxins. The cyanobacteria produce toxins which help them survive in their environment by protecting from predators and providing advantages during stressful periods yet these toxins accumulate in water organisms leading to health risks in drinking water supply systems [119] (Table 2).

### 3.2. Anatoxin-a

Anatoxin-a functions as an acetylcholine receptor agonist to disrupt nervous system operations and causes muscle paralysis and respiratory failure. The toxin appears in mat formation at its initial stages and scientists believe this biological response helps microorganisms adapt to environmental challenges or acquire new substrate territories. The toxicological research identifies Microcoleus autumnalis as one species which produces anatoxin-a [120].

### 3.3. Microcystins (MCs) and Nodularins (NODs)

The hepatotoxic compounds Microcystins (MCs) and Nodularins (NODs) block protein phosphatases to damage liver cells and may result in tumor development. The production of microcystins occurs in various benthic organisms and nodularins are less abundant yet equally significant. The toxin-producing activity of benthic microorganisms intensifies under stressful environmental conditions that include scarce nutrients and intense light conditions [121] (Table 2).

### 3.4. Saxitoxins (STXs)

The blockage of sodium channels by Saxitoxins (STXs) stops the transmission of nerve impulses. The aquatic species Microseira wollei, among others, produces these substances in North American waters. Monitoring benthic mats presents challenges to scientists because toxin production varies across geographic areas [122].

### 3.5. Cylindrospermopsins (CYNs)

The cytotoxin Cylindrospermopsins (CYNs) stops protein synthesis while damaging multiple body organs. The main distribution of CYN-producing benthic cyanobacteria occurs in Australian water systems. The presence of CYN-producing cyanobacteria in benthic mats presents additional water management complications because these toxins survive for long periods in the environment [123].

### 3.6. Factors Affecting Toxin Production

The production of toxins by benthic cyanobacteria shows high variability because it depends on environmental conditions and genetic composition of the bacteria: Low concentrations of phosphorus or nitrogen in the environment stimulate benthic cyanobacteria to increase their toxin quantity in each cell [124]. The mats that form consist of toxic and non-toxic strains of organisms. Toxic strain composition within the mat directly impacts the total toxicity measured in the mat [125]. The production of toxins by benthic cyanobacteria depends on multiple environmental stressors which include light intensity together with temperature fluctuations and competition from other microorganisms [126].

### 3.7. Taste and Odor (T&O) Compounds

Benthic cyanobacteria create toxic substances in addition to releasing compounds that affect drinking water taste and odor which results in complaints from consumers despite no health hazards.

### 3.8. Geosmin and 2-Methylisoborneol (MIB)

The two most frequently detected T&O metabolites exist as Geosmin and 2-Methylisoborneol. (Table 2). Water develops earthy or musty odor at extremely low concentration levels (parts per trillion). The main producers of these compounds in benthic environments are cyanobacteria while actinobacteria participate in lower levels of production [16]. Table 2 details the environmental factors driving cyanotoxin and T&O compound production, emphasizing the role of nutrient stress and temperature in exacerbating benthic cyanobacterial impacts.

**Table 2:** Key cyanotoxins and taste and odor compounds produced by benthic cyanobacteria: The following table summarizes the key cyanotoxins and taste and odor compounds produced by benthic cyanobacteria, their effects, and the environmental factors that influence their production.

Compound	Type	Effects	Environmental Influences
Anatoxin-a	Neurotoxin	Disrupts nervous system, causing muscle paralysis and respiratory failure.	Produced during early stages of mat formation, influenced by nutrient fluctuations and competition [127].
Microcystins (MCs)	Hepatotoxins	Inhibit protein phosphatases, leading to liver cell damage and potential tumor formation.	Increased production under nutrient stress, particularly in oligotrophic conditions [128].
Nodularins (NODs)	Hepatotoxins	Similarly to microcystins, targeting liver cells and promoting cellular damage.	Less common, but produced under environmental stress, including nutrient limitation [129].
Saxitoxins (STXs)	Neurotoxin	Block sodium channels, preventing nerve impulse conduction.	Geographic variability, with higher production in certain species under environmental stress [130].
Cylindrospermopsins (CYNs)	Cytotoxins	Inhibit protein synthesis, causing damage to multiple organs.	Found primarily in Australian systems; influenced by nutrient availability and temperature [131].
Geosmin	Taste and Odor (T&O)	Imparts an earthy or musty odor to water, even at very low concentrations.	Strongly correlated with warm temperatures, elevated in floating mats [16].
2-Methylisoborneol (MIB)	Taste and Odor (T&O)	Causes musty or earthy odors in water.	Elevated in warmer temperatures, with higher concentrations in floating mats [132].

### 3.9. Dominance in Benthic Mats

Research findings show that cyanobacteria produce T&O compounds as the primary benthic mat producers, although actinobacteria function as supplementary producers. The dominance of cyanobacteria requires water utilities to understand its importance because traditional monitoring methods could miss benthic sources when assessing planktonic cyanobacteria [133,134].

### 3.10. Environmental Influences

Warm temperature conditions promote cyanobacterial metabolic activity which directly affects the production of geosmin and MIB. The surface position of floating mats results in increased T&O compound concentrations because water surface conditions create optimal conditions for photosynthesis and nutrient uptake [135].

### 3.11. Impact on Water Quality and Public Perception

Geosmin and MIB do not threaten human health, but they profoundly diminish public trust in water supply quality. A musty smell or taste in drinking water creates a perception of danger in consumers even though the water meets all the established health-based requirements. The successful detection and handling of T&O events is crucial to protect public water trust [31].

### 3.12. Monitoring and Early Detection

Modern molecular assays that use quantitative polymerase chain reaction (qPCR) to detect biosynthesis genes can provide advance notice of geosmin and MIB production and toxin formation. The tools provide utilities with the capability to detect T&O or toxin issues before they become noticeable to consumers or cause health risks [136].

## 4. Interactions Between PET Microplastics and Benthic Cyanobacteria

Microplastics based on polyethylene terephthalate (PET) have added two types of stressors to benthic microbial communities in freshwater ecosystems especially affecting cyanobacteria. [Table 3] Benthic cyanobacteria, which act as primary biofilm builders in riverbeds and lake bottoms, develop physical, biological, and chemical connections with PET microplastics which alters their ecological functions as well as PET microplastic behavior within aquatic environments [137,138]. Benthic and planktonic cyanobacteria exhibit distinct responses to PET microplastics and leachates due to differences in morphology, ecological niche, and physiology. Table 3 compares these responses, highlighting how benthic cyanobacteria’s filamentous structure and EPS production enhance PET adhesion and leachate assimilation, while planktonic species are more susceptible to photosynthetic disruption and toxin suppression.

### 4.1. ***Colonization of PET Microplastics by Benthic Biofilms***

PET microplastics lose their inert nature after their entrance into freshwater environments. Microorganisms quickly establish colonies on the surfaces of PET which creates “plastisphere” microbial communities. The filamentous structure of benthic cyanobacteria combined with their EPS production enables these organisms to become principal colonizers of plastics [144,145].

The colonization process depends on multiple factors which determine its outcome.

➢ PET microplastics have surface characteristics that enable bacteria to adhere to them because they are rough and hydrophobic [146].
➢ PET particles experience surface roughness increases because of UV radiation and mechanical abrasion which leads to enhanced microbial attachment [147].
➢ The EPS production of benthic cyanobacteria functions as biological adhesive material which captures PET microplastics into forming mats [148].

The colonized plastics enter biofilms where they become fully integrated within the complex three-dimensional matrix which consists of cyanobacteria combined with bacteria and fungi and detritus [146].

### 4.2. ***Retention, Stabilization, and Transport Dynamics***

Benthic cyanobacterial mats with their adhesive mucilaginous composition act as essential agents that trap and immobilize PET microplastics found in river sediments. This process is supported by multiple mechanisms.

- Biofilms modify hydrodynamic conditions by modifying boundary layer characteristics which promotes suspended particles including PET fragments to settle down [149].
- PET microplastics become physically trapped inside the mat structure through physical entrapment [150].
- The aged PET particles develop negative surface charges which create electrostatic bonds with EPS components [146].

PET particles embedded in the mat structure penetrate deeply into the structure when flow rates are low which results in prolonged retention on riverbeds. The mechanical actions caused by high-flow floods break down river mats which makes microplastics detach from their location and travel further downstream. Biofilm clumps that detach from their surfaces transport PET particles as vectors that disperse microplastics across long distances.

### 4.3. ***Ecotoxicological Implications for Benthic Cyanobacteria***

Cyanobacterial mat ecosystems experience active changes in their physiological and ecological functions because of PET microplastic integration.

1. Physical Disruption:

The integration of PET particles within biofilms produces two effects on mat structure. The presence of physical shading elements created by PET microplastics blocks sunlight from reaching the surface resulting in reduced photosynthesis rates and PET microplastics have the ability to break down EPS matrices which might result in weakened mats [151].

2. Chemical Stress:

The leaching of phthalates, bisphenols, and flame retardants occurs from PET microplastics particularly after enduring environmental degradation [152]. PET surfaces have the ability to attract environmental pollutants that include heavy metals (e.g., lead and cadmium); hydrophobic organic pollutants (e.g., PAHs and PCBs); and antibiotics and pharmaceuticals [153]. The pollutants introduce stress that damages membranes and disrupts metabolism in cyanobacteria leading to lower primary productivity and modified mat structure.

3. Selective Pressure and Community Shifts:

Microplastic-associated pollutants apply pressure on benthic microbial communities which allows stress-tolerant or opportunistic taxa to thrive while sensitive species decline. This can lead to shifts in species richness and evenness; the dominance of toxin-producing cyanobacteria; and changes in ecological functions such as nitrogen fixation [154].

4. Alterations in EPS Composition:

The production of EPS by benthic cyanobacteria can change when they experience the stress of microplastics. Benthic cyanobacteria boost their protection against stress by increasing the number of EPS they produce. Benthic cyanobacteria may enhance their protection through two EPS modifications including quantity increases and chemical composition alterations that improve particle binding. These alterations modify the natural processes of nutrient cycling as well as sediment stability and the way microbial and macroinvertebrate communities interact with each other [155].

EPS biosynthesis involves complex metabolic pathways, primarily the synthesis of polysaccharides via the Wzy-dependent pathway, where glycosyltransferases assemble sugar units into extracellular polymers [156]. Key enzymes, such as UDP-glucose pyrophosphorylase and cellulose synthase, regulate EPS production, with genes like wcaG and bcsA upregulated under pollutant stress [157]. PET leachates, particularly phthalates, may trigger transcriptional activation of EPS-related genes via sigma factors, enhancing mucilage production to mitigate oxidative stress. These changes alter nutrient cycling by increasing sediment cohesion and modify microbial interactions by favoring stress-tolerant species within the plastisphere [158]. However, excessive EPS production may reduce mat porosity, limiting oxygen and nutrient diffusion, which could impair cyanobacterial growth [159].

## 5. Impacts on Nutrient Cycling and Contaminant Dynamics

The presence of PET microplastics in benthic cyanobacterial mats leads to substantial changes in freshwater ecosystem biogeochemical processes: PET particles function as localized nutrient accumulation points which alter the availability of nutrients inside mats. The modification of essential microbial functions including nitrification, denitrification, and phosphorus solubilization would result in changes to the overall ecosystem nutrient distribution [160]. Benthic mats containing PET along with pollutants act as storage units and transmission pathways for contaminant distribution which affects pollution patterns in the environment. The feeding habits of insect larvae, as well as snails and fish, create a pathway for microplastics to enter the aquatic food web because these organisms accidentally consume microplastics while eating benthic mats [46].

## 6. Knowledge Gaps and Research Priorities

Research about microplastic–biofilm interactions continues to advance yet scientists still lack essential information about the effect of PET microplastics on benthic cyanobacteria. PET-derived chemicals have unidentified mechanisms which affect cyanobacterial metabolic functions and EPS production. The sensitivity of PET microplastics to different cyanobacterial species remains unclear because specific research on this topic is insufficient. The extended effects of PET exposure on benthic mat stability and ecosystem functioning during long durations need additional research because most experiments only last short periods [161]. Field-level experiments should verify laboratory results by evaluating PET microplastic effects under conditions that combine multiple environmental stressors including temperature and UV radiation and nutrient availability. Future investigations should unite field monitoring with laboratory research and modern molecular approaches like metagenomics and metabolomics to elaborate on the complex PET microplastic–benthic cyanobacterial ecosystem relationships [162].

Contradictory findings on whether PET leachates promote or inhibit cyanobacterial growth and toxin production highlight a critical research gap. Factors such as leachate concentration, exposure duration, and cyanobacterial species specificity need systematic investigation. For example, *Oscillatoria* may exhibit greater tolerance to PET leachates than Microcystis due to its filamentous structure, but direct comparisons are lacking [20].

## 7. Molecular Responses of Benthic Cyanobacteria to PET and PET Leachates

Benthic cyanobacteria maintain a vital position in aquatic ecosystems as they live within sedimentary environments. Benthic cyanobacteria play a vital role by cycling nutrients while producing primary matter and sustaining the food chain structure. The rising numbers of PET microplastics together with PET leachates in aquatic ecosystems cause severe impacts on molecular processes [119]. Cyanobacteria develop various stress responses after being exposed to these contaminants that become visible at their molecular level. The molecular effect of PET exposure leads to four main functional changes in cyanobacteria involving gene expression modifications of growth and metabolic functions, oxidative stress responses, toxin and secondary metabolite production regulation, and signaling pathway alterations [163]. The following sections delve deeper into these molecular mechanisms.

### 7.1. Gene Expression Related to Growth and Metabolism

Exposure to PET microplastics and leachates altered gene expression in benthic cyanobacteria, affecting growth and metabolism. The most immediate effect on benthic cyanobacteria involves reduced growth speeds because of PET leachate toxicity and mechanical limitations from microplastics. Cellular replication mechanisms are disrupted as part of the stress response leading to modified gene expression patterns of growth-related genes [20]. The cells downregulate protein synthesis and cell division genes during stressful periods because they want to conserve energy and resources.

**Figure 3:**
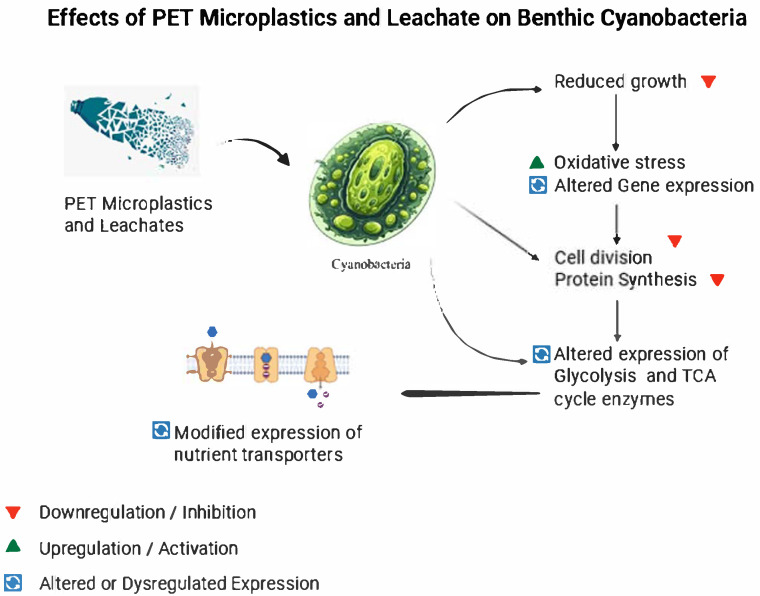
Schematic overview of the effects of PET microplastics and leachates on benthic cyanobacteria, highlighting growth inhibition, metabolic reprogramming, and nutrient uptake alterations.

The metabolic activities of benthic cyanobacteria change when they encounter PET leachates. The expression patterns of central carbon metabolism enzymes including glycolysis and tricarboxylic acid (TCA) cycle enzymes change in response to PET leachates [164]. The stress adjustments made by cyanobacteria involve redistributing metabolic pathways which help sustain their cellular operations when faced with unfavorable environmental situations. Research shows that benthic cyanobacteria modify their essential nutrient metabolism patterns. The environmental changes in sediment due to PET contamination led to modified nutrient conditions which trigger cyanobacteria to modify their nutrient uptake systems [165] (Figure 3).

### 7.2. Oxidative Stress and Antioxidant Defense Pathways

PET leachates trigger oxidative stress reactions in benthic cyanobacteria when they come into contact, which is a typical stress response to chemical pollutants [100]. The leachates contain multiple organic compounds which produce reactive oxygen species (ROS) during their degradation process or when they encounter cyanobacterial cells [166]. ROS molecules inflict substantial damage to cell membranes, proteins, and DNA components. Benthic cyanobacteria activate their defense mechanisms to counteract the harmful effects of oxidative stress [167]. The cell activates its defense mechanisms by elevating antioxidant protection systems. The elevated ROS levels trigger the cells to boost the production of antioxidant enzymes which include superoxide dismutase (SOD), catalase, and glutathione peroxidase [168]. Enzymes perform vital functions by breaking down dangerous ROS molecules to protect cellular parts from oxidative damage. Additionally, the antioxidant molecules glutathione and vitamin C actively work with free radical scavenging alongside the maintenance of cellular redox balance [169].

Redox signaling functions as a vital part of cyanobacterial oxidative stress responses in addition to enzymatic detoxification mechanisms. ROS accumulation sets off signaling pathways that activate transcription factors like such as PerR, or two-component systems like Hik33, to regulate antioxidant and stress response genes. In *Synechocystis* sp. PCC 6803, PerR (slr1738) senses H_2_O_2_ via metal-catalyzed oxidation, derepressing genes like isiA and sll1483 to combat oxidative stress [170]. Through redox-sensitive regulation, the cyanobacteria adapt to long-term PET leachate exposure and protect their cellular structure even when facing continuous environmental stress [161].

### 7.3. Regulation of Toxin and Secondary Metabolite Biosynthesis

A range of bioactive secondary metabolites, such as microcystins (which harm the liver), anatoxins (which affect the nervous system), and Nodularins, are generated by cyanobacteria to protect them from environmental stressors, such as PET microplastics and their byproducts, including BPA and phthalates [20,171]. The synthesis of these compounds is controlled by enzymes called nonribosomal peptide synthetase (NRPS), polyketide synthase (PKS), and gene clusters called mcy, sxt, and ana are responsible for their production [171,172,173]. The mcy cluster in Microcystis aeruginosa and benthic cyano-bacteria (*Oscillatoria* sp. and *Pseudanabaena* sp.) includes mcyD (PKS), mcyA–C (NRPS), and mcyE genes that build microcystins with malonyl–CoA and amino acids (glutamate and aspartate) [171]. Study shows that PET microplastics and leachates at this level cause Microcystis aeruginosa to increase mcyD expression which is triggered by ROS created by BPA and other compounds in the leachate [174]. When ROS cause damage to cells, the upregulation of genes helps microcystin be produced to protect the cell membrane, proteins, and DNA [175].

The regulation of toxin biosynthesis includes transcription factors and signaling pathways that react to changes in the environment such as oxidative stress, limited nutrients, and different light conditions [176,177]. In *Synechocystis* sp. PCC 6803, the PerR (slr1738) transcription factor is activated by H_2_O_2_ and iron which helps express the isiA (iron-stress-induced protein) gene which might control mcy expression by changing the cell’s redox balance [170]. For example, SigE will react to stresses (such as high light or nutrient problems) and boost mcy promoter activity to increase toxin gene expression [178]. Researchers using proteo-mic techniques have discovered that under PET exposure, PKS/NRPS enzymes are expressed more, thanks to increased levels of acetyl–CoA carboxylase and glutamine synthetase which make more malonyl–CoA and amino acids available [20]. PET microplastics boost EPS, keeping biofilms stable and holding onto nutrients (like nitrogen and phosphorus) which helps both precursor manufacture and the production of toxins [179].

### 7.4. Signal Transduction Pathways and Quorum Sensing

Benthic cyanobacteria use signal transduction to detect PET microplastics and leachates and respond by creating toxins and secondary metabolites [180]. Using Hik33 as an example, two-component systems can detect ROS, changes in nutrients or light, and then use phosphorylation to change the activity of response regulators, such as Rre1 and affect gene activity [180]. As a result, PET leachate ROS led to Hik33 phosphorylation which raises the activity of mcyD and photosynthetic genes (psbA, rbcL), supporting higher toxin production and growth in *Oscillatoria* sp. and *Pseudanabaena* sp. [20]. During oxidative stress, thioredoxin changes enzyme activity which may affect the attachment of transcription factors to the mcy promoter [20]. When exposed to PET, proteomic studies uncover that kinases and phosphatases become more active and this modifies glycolysis and the TCA cycle, ensuring that PKS/NRPS systems have the building blocks they need [170].

QS is responsible for controlling how cyanobacteria respond to their population size, guiding biofilm creation, movement, and metabolite production [181]. Although AHLs are well known in heterotrophic bacteria, cyanobacteria might use AHL-like substances or other autoinducers to manage the expression of mcy and ana genes [182]. BPA in PET leachates can impact QS by blocking the activity of LuxI homologs and therefore change the amount of toxins produced [183]. As an example, exposure to BPA can cause Microcystis to produce different biofilm amounts, possibly by messing with how the bacteria detect their autoinducers and how they express mcy. When benthic areas are low in nutrients, QS stimulates EPS production which makes the community more resilient and able to keep nutrients [179]. Even so, the particular QS molecules and how they react with PET leachates are not well understood; more research in transcriptomics and proteomics is required.

## 8. Implications for Ecosystem Health and Water Quality

The molecular responses of benthic cyanobacteria to PET microplastics and leachates not only affect the organisms themselves but also have broader implications for ecosystem health and water quality. The ecological impacts of PET contamination extend across trophic levels, with potential risks for bioaccumulation, trophic transfer, and water quality. The following sections detail these critical issues.

### Bioaccumulation and Trophic Transfer Risks

PET microplastics present a major environmental concern because they have the ability to build up in aquatic food chains. Benthic organisms consume microplastics which primarily include PET particles and use cyanobacteria as their food source which continues up the food chain to higher trophic levels. PET contaminants that build up in cyanobacteria cells transfer to herbivores when these bacteria are eaten. The movement of pollutants through the food chain allows them to reach predators starting from fish up to birds and humans [184].

PET microplastics and their leachates building up in aquatic life can greatly harm the environment, mainly due to trophic transfer [185]. Microplastics have been discovered in the stomachs of freshwater fish (*Oreochromis niloticus*) [186], bivalves (*Dreissena polymorpha*) [187], and zooplankton (*Daphnia magna*) species [188]. Toxic particles may collect in tissues, disrupt how animals eat, and slow down their development. Moreover, substances in plastic that are related to BPA can pass through epithelial membranes and upset the function of hormone signals. It has been proven that PET leachates are taken up by algae and then passed on to grazers, showing a clear route of exposure [189]. Using such information tells us why PET pollution is harmful to the environment.

The presence of PET microplastics and leachates within aquatic organisms creates multiple potential risks for these organisms. Plastics persist across environmental timescales so bioaccumulation of plastic in food webs enables continuous exposure of organisms across different trophic levels [190]. PET leachates release multiple toxic substances which disrupt organisms’ health through endocrine disruption and carcinogenic substance releases. The accumulation of these chemicals in higher organisms results in physiological damage and reproductive problems that generate lasting ecological effects [191]. PET contaminants transferred through feeding relationships will alter the relationships between predators and prey as well as affect species numbers and ecosystem patterns. Organisms which fail to eliminate microplastics or detoxify leachates will see lower reproductive success and reduced survival that causes population structure changes and endangers biodiversity [48].

## 9. Challenges for Drinking Water Treatment

The occurrence of PET microplastics in freshwater water bodies presents major issues that interfere with drinking water treatment technologies. The classic water treatment methods—coagulation, filtration, and disinfection—fail to effectively eliminate microscopic particles. The small dimensions of PET microplastics enable them to pass through standard filtration systems while remaining in treated water which creates health risks [192]. PET microplastics create additional challenges to water treatment because they release dangerous chemicals into the water system. Standard water treatment methods struggle to eliminate toxic degradation byproducts of PET and production-related additives like plasticizers, flame retardants, and stabilizers from leachates. The double harm of chemical agents and physical particles in water shows the necessity to develop better water purification approaches [22]. Water treatment technologies using activated carbon filtration, reverse osmosis, and advanced oxidation processes show microplastic removal ability but remain too expensive for widespread implementation [193]. Research needs to continue as it aims to create affordable solutions that can effectively tackle the problems caused by PET microplastics and their leachates in drinking water systems.

## 10. Monitoring and Mitigation Strategies

Effective monitoring and control methods are needed to deal with the increasing problem of PET pollution in aquatic environments. Continuous monitoring of microplastic concentrations in water bodies, sediments, and biota is needed to determine the level of contamination and to follow the trends over time. This monitoring should not only be focused on the presence of PET particles but also include analysis of leachates and associated toxic compounds, which can be a source of great risk to aquatic life and human health [194,195]. Mitigation strategies for minimizing PET pollution in aquatic systems need a multi-faceted approach. One of the best practices is decreasing the amount of PET litter entering water bodies. This could be achieved through better waste management practices—higher recycling rates and biodegradable substitutes for PET plastics [196]. Public awareness campaigns and policy actions promoting the decrease in single-use plastics are also essential in addressing the source of PET pollution [22].

To control PET microplastics and leachates, we must use a mix of modern sensors, break-down methods, and regulatory rules. Some new approaches use enzymes called PETase and MHETase, taken from *Ideonella sakaiensis*, to break PET into harmless monomers [197,198]. New discoveries in biotechnology are examining groups of microbes and modified strains that can degrade PET at room temperature. On the regulatory side, the EU’s REACH legislation is now looking at microplastic additives and the substances they release for restriction and detailed evaluation. Moreover, new methods using remote sensing and machine learning are created to detect microplastics in water immediately. To deal with the ecological hazards of PET pollution, nations need to cooperate on both new solutions and rules for using PET.

Biodegradation research of PET plastics combined with the development of bioremediation techniques might provide solutions to reduce the current impact of microplastic pollution. Some microorganisms and enzymes have been shown to have potential in decomposing PET plastics, and these approaches could be looked into as part of bigger-scale cleanup efforts [199,200]. Ultimately, the integration of interdisciplinary research methods, including remote sensing, molecular biomarkers, and predictive models, will provide a better understanding of the long-term ecological consequence of PET pollution. These types can guide decision making and the development of effective management practices for maintaining ecosystem health and water quality.

## 11. Conclusions

The widespread environmental distribution of PET microplastics and leachates is a multiple risk to aquatic ecosystems, especially in interactions with benthic cyanobacteria. Growth, metabolism, and stress tolerance of cyanobacteria are altered by PET and its degradation products, as indicated by the emerging evidence, affecting key molecular pathways. These interactions may be responsible for the improved survival and growth of cyanobacteria in polluted environments, which could lead to ecosystem instability and harmful algal blooms. Further interdisciplinary work is required to completely understand the molecular mechanisms and to give advice to abate plastic pollution and its biological impacts.

## Figures and Tables

**Table 1:** Overview of PET microplastics: sources, aging, and leachate impacts.

Aspect	Key Points	Details/Examples	Reference
Sources and Distribution of PET Microplastics	Origin and environmental dispersion of PET microplastics	Primary sources: microbeads, pellets; Secondary: fragmentation of larger debris; Pathways: improper disposal, runoff, industrial discharge, WWTPs; Found in water, sediments globally	[38]
Environmental Aging and Surface Modification of PET	Degradation processes and enhanced ecological risks	Factors: UV radiation, mechanical abrasion, hydrolysis, bioactivity; Effects: increased roughness, biofilm formation, chemical release	[116]
PET Leachates: Composition and Environmental Relevance	Chemical makeup and toxicological impact	Components: TPA, BHET, phthalates, metals; Effects: photosynthesis inhibition, oxidative stress, endocrine disruption; Risk from complex chemical mixtures	[117]

**Table 3:** Comparison of PET microplastic and leachate effects on planktonic and benthic cyanobacteria.

Aspect	Planktonic Cyanobacteria	Benthic Cyanobacteria	References
Morphology	Unicellular or small colonies (e.g., Microcystis, ~3.6 μm)	Filamentous, mat-forming (e.g., *Oscillatoria*, >180 μm)	[139]
PET Adhesion	Limited due to small size and lack of EPS matrix	Enhanced by EPS and filamentous structure, promoting plastisphere formation	[140]
Photosynthetic Impact	High sensitivity to shading by microplastics	Moderate sensitivity; adapted to low-light benthic environments	[141]
Toxin Production Response	Variable; often inhibited by high leachate concentrations (e.g., mcyD downregulation)	Upregulation of toxin genes (e.g., mcyD 1.8-fold increase) at low concentrations	[142]
Ecological Role	Primary producers in water column; bloom formation	Nutrient cycling in sediments; mat stabilization	[143]
Leachate Metabolism	Limited assimilation of PET monomers due to short exposure times	Potential use of monomers as carbon sources in stable benthic mats	[19]

## Data Availability

All data connected with this research study are presented in the manuscript.
